# Microvesicles from malaria-infected red blood cells activate natural killer cells via MDA5 pathway

**DOI:** 10.1371/journal.ppat.1007298

**Published:** 2018-10-04

**Authors:** Weijian Ye, Marvin Chew, Jue Hou, Fritz Lai, Stije J. Leopold, Hooi Linn Loo, Aniruddha Ghose, Ashok K. Dutta, Qingfeng Chen, Eng Eong Ooi, Nicholas J. White, Arjen M. Dondorp, Peter Preiser, Jianzhu Chen

**Affiliations:** 1 School of Biological Sciences, Nanyang Technological University, Singapore; 2 Singapore-MIT Alliance for Research and Technology, Infectious Disease Interdisciplinary Research Group, Singapore; 3 Humanized Mouse Unit, Institute of Molecular and Cell Biology, Agency of Science, Technology and Research, Singapore; 4 Mahidol Oxford Tropical Medicine Research Unit, Faculty of Tropical Medicine, Mahidol University, Bangkok, Thailand; 5 Centre for Tropical Medicine and Global Health, Nuffield Department of Medicine, University of Oxford, Oxford, United Kingdom; 6 Department of Internal Medicine, Chittagong Medical College Hospital, Chittagong, Bangladesh; 7 Duke-National University of Singapore Medical School, Singapore; 8 Koch Institute for Integrative Cancer Research and Department of Biology, Massachusetts Institute of Technology, Cambridge, MA, United States of America; London School of Hygiene and Tropical Medicine, UNITED KINGDOM

## Abstract

Natural killer (NK) cells provide the first line of defense against malaria parasite infection. However, the molecular mechanisms through which NK cells are activated by parasites are largely unknown, so is the molecular basis underlying the variation in NK cell responses to malaria infection in the human population. Here, we compared transcriptional profiles of responding and non-responding NK cells following exposure to *Plasmodium*-infected red blood cells (iRBCs) and identified MDA5, a RIG-I-like receptor involved in sensing cytosolic RNAs, to be differentially expressed. Knockout of MDA5 in responding human NK cells by CRISPR/cas9 abolished NK cell activation, IFN-γ secretion, lysis of iRBCs. Similarly, inhibition of TBK1/IKKε, an effector molecule downstream of MDA5, also inhibited activation of responding NK cells. Conversely, activation of MDA5 by liposome-packaged poly I:C restored non-responding NK cells to lyse iRBCs. We further show that microvesicles containing large parasite RNAs from iRBCs activated NK cells by fusing with NK cells. These findings suggest that NK cells are activated through the MDA5 pathway by parasite RNAs that are delivered to the cytoplasm of NK cells by microvesicles from iRBCs. The difference in MDA5 expression between responding and non-responding NK cells following exposure to iRBCs likely contributes to the variation in NK cell responses to malaria infection in the human population.

## Author summary

Malaria is an important parasitic disease with a major public health concern. Malaria pathogenesis involves a complex interplay between parasitic and host factors. A better understanding of early host response and the determinants of immunity are essential to developing innovative therapeutic approaches. Natural killer (NK) cells are important immune cells in protection against malaria infection but show significant differences in their responses in the human population. Here we analyze the differences between human NK cells that respond to and don’t respond to malaria infection. We found that human NK cells that respond to malaria-infected red blood cells (iRBC) have higher levels of a pathogen recognition receptor, MDA5. This receptor is activated by small vesicles released from iRBC. By activating MDA5 with a small molecule agonist, we can improve non-responder NK cells to clear iRBC. Our study provides new insights into the mechanism by which NK cells control malaria infection and possible NK cell-based intervention of malaria infection in human.

## Introduction

Human malaria is caused by parasites of the genus *Plasmodium*, and of which, *P*. *falciparum* causes most cases of severe malaria. The host innate immune system is the first line of defense against *Plasmodium* infection, and the outcome of early host-parasite interaction is a strong determinant for later immunopathology and adaptive immune responses [1]. Natural killer (NK) cells, a key cell type of innate immunity, play a critical role in limiting acute malaria infection by both cell-mediated cytotoxicity and IFN-γ secretion [2]. In murine models, malaria infection leads to a rapid proliferation of NK cells [3], and depletion of NK cells results in higher parasitemia and accelerated disease progression [4–6]. In malaria-infected children, elevated NK cell counts and increased NK cell cytotoxicity are correlated with lower parasitemia [7]. Similarly, elevated NK cell counts are observed in adult malaria patients [8]. NK cells can directly lyse *Plasmodium*-infected red blood cells (iRBCs) [9, 10] and are also one of the earliest sources of IFN-γ and soluble granzyme following experimental infection of malaria-naïve volunteers with *P*. *falciparum* [11]. Furthermore, a single *P*. *falciparum* challenge is sufficient to induce lasting NK cell responses [12].

Innate immune cells, such as NK cells, recognize pathogens through pattern-recognition receptors (PRR). Studies have shown that activation of human macrophages and dendritic cells (DCs) by iRBCs requires Toll-like receptors (TLR) and RIG-I-like receptors (RLR), which includes RIG-1, MDA5, and LGP2. Engagement of TLR2 and TLR4 by *P*. *falciparum* glycosylphosphatidylinositol (GPI) stimulates macrophages and DCs to secrete TNF-α [13, 14]. Parasitic DNA and RNA also activate DCs via TLR9 [15] and MDA5 [16], respectively. In contrast, TLRs are individually dispensable for NK cell responses to malaria infection in mice [17]. Although NKp30 has been shown to bind PfEMP1 [18], the role of PfEMP1 in NK cell activation remains controversial because NK cells can still be activated by parasite that does not express surface PfEMP1 [19] and blocking of NKp30, NKp44 or NKp46 with antibodies does not affect NK cell control of parasitemia *in vitro* [10]. To date, human NK cell activation by iRBCs was shown to require cell-cell contact involving cell adhesion molecules such as LFA-1 [10], but the nature and identity of parasite molecular pattern and the specific PRR involved are largely unknown.

Interestingly, NK cell responses to malaria infection vary significantly in the human population. For example, one study reported an average of 2.4% of NK cells are IFN-γ-positive in mild malaria patients [8], whereas a different study reported an average of 14% of IFN-γ-positive NK cells [12]. Variations in NK cell responses to iRBCs *in vitro* are also observed. Based on percentages of NK cells that express IFN-γ, donors have been classified as responders or non-responders [20]. The observed heterogeneity in NK cell responses to malaria infection has been attributed to differences in NK cell receptor expression, such as killer cell immunoglobulin-like receptors (KIR) [20], and lifestyle factors, such as smoking and alcohol use [21, 22]. The molecular basis underlying the variations of NK cell responses to malaria infection has not been fully investigated.

In this study, we investigated the nature and identity of parasite component and NK cell receptor that are involved in human NK cell responses to malaria infection. By comparing gene expression between NK cells from responders and non-responders, we identified MDA5, an RLR for sensing cytosolic RNA, as being differentially expressed. MDA5 is critical for NK cell responses to malaria infection as inhibition of MDA5 in responder NK cells abolishes NK cell responses to iRBCs and activation of MDA5 in non-responder NK cells restores NK cell responses to iRBCs. We further show that RNA-containing microvesicles released from iRBCs can activate NK cells by fusing with NK cells. Our study thus shows that parasite RNA-containing microvesicles from iRBCs and MDA5 in NK cells are involved in NK cell sensing of malaria infection and activation of NK cell responses. The difference in MDA5 expression between NK cells from responders and non-responders is likely a significant factor that contributes to the variation in NK cell responses to malaria infection in the human population.

## Results

### NK cell responses to iRBCs are heterogeneous in human population

**Fig 1:**
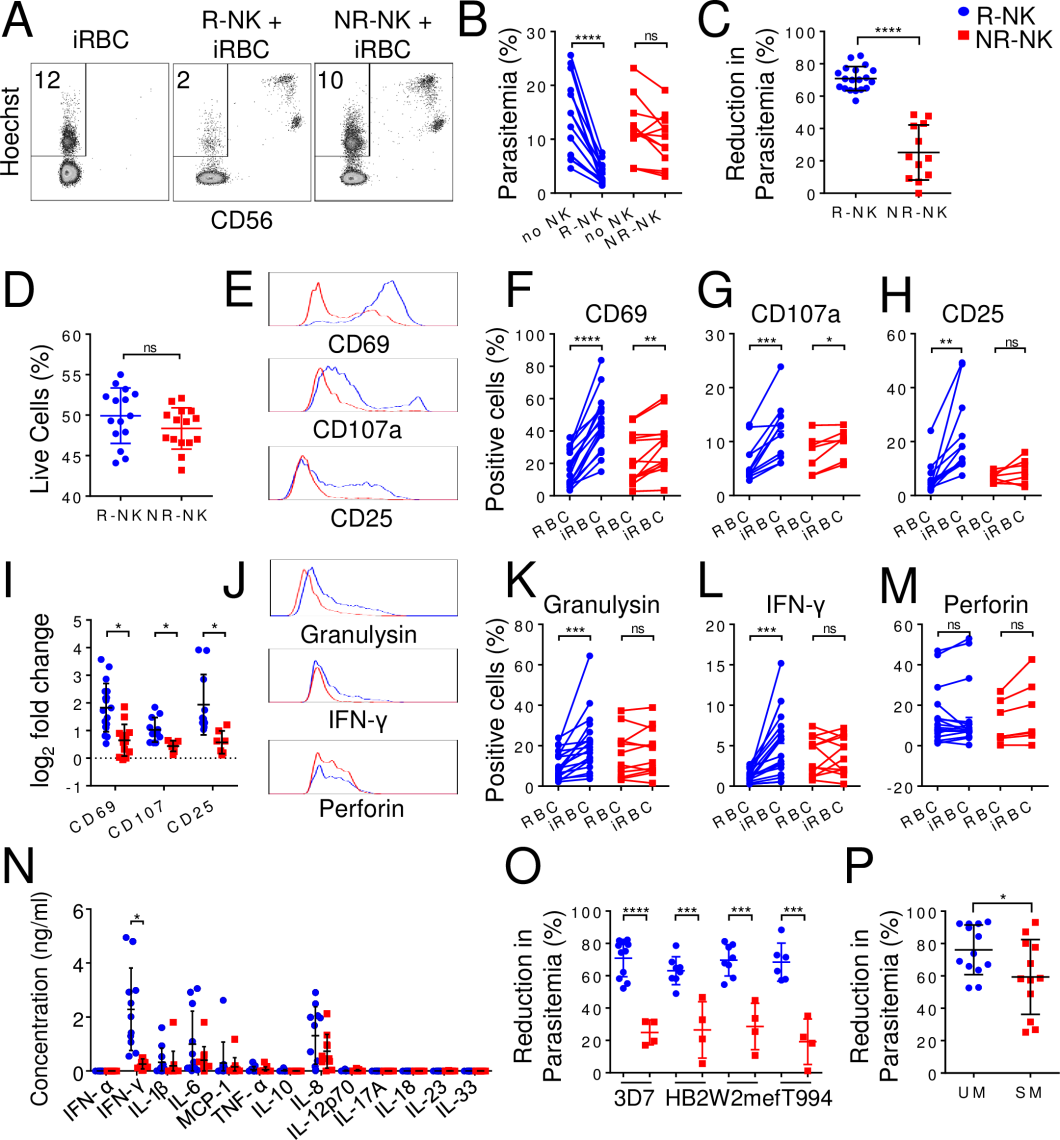
Variation of NK cell responses to iRBCs among different individuals. Human NK cells were purified (>95%) from fresh blood and co-cultured with iRBCs for 96 h. Parasitemia and the expression of various NK cell surface markers and intracellular proteins were assayed by flow cytometry. The level of cytokines in the culture supernatants was quantified by multiplex immunoassay at 48 h. **(A)** Representative CD56 vs. Hoechst staining profiles of iRBCs alone, iRBCs co-cultured with responder NK cells (R-NK) or with non-responder NK cells (NR-NK). The number represents the percentage of iRBCs (parasitemia). CD56-positive cells are NK cells. **(B)** Paired plots of parasitemia in the absence of NK cells (No NK) and with either R-NK or NR-NK. **(C)** Comparison of reduction in parasitemia in the presence of either R-NK (blue) or NR-NK (red) cells from different malaria-naïve individuals. **(D)** Percentage of live (DAPI-negative) NK cells after 96 hrs of co-culture. **(E)** Representative histograms comparing CD69, CD107 and CD25 expression on R-NK and NR-NK cells from one responder (blue trace) and one non-responder (red trace). **(F-H)** Paired plots showing changes in the percentage of NK cells positive for CD69 **(F)**, CD107a **(G)** and CD25 **(H)** following co-culture with either RBC or iRBC. **(I)** Comparison of log_2_ fold change in expression levels of CD69, CD107 and CD25 between R-NK and NR-NK cells following co-culture with iRBC. **(J)** Representative histograms comparing granulysin, IFN-γ and perforin expression on R-NK and NR-NK cells from one responder and one non-responder. **(K-M)** Paired plots showing changes in the percentage of NK cells positive for granulysin **(K)**, IFN-γ **(L)** and perforin **(M)** following co-culture with either RBC or iRBC. **(N)** Comparison of soluble mediators in the culture supernatants. **(O)** Comparison of reduction in parasitemia following co-culture of R-NK or NR-NK cells in the presence of different strains of parasites. R-NK and NR-NK cells were co-cultured with the indicated parasite strain for 96 h. Parasitemia was quantified by flow cytometry. **(P)** Comparison of reduction in parasitemia in the presence of NK cells from either uncomplicated malaria (UM) patients or severe malaria SM patients. NK cells were purified from frozen buffy coat and co-cultured with 3D7-infected RBCs for 96 h and parasitemia was quantified by flow cytometry. Each symbol in B-D, F-I, K-P represents a different individual. Joined lines show experimental pair. Error bars represent mean ± SD. * p<0.05, ** p<0.01, *** p<0.001, **** p<0.0001, ns: not significant.

In our study of NK cell responses to iRBCs, we noticed differences in the control of parasitemia by NK cells from different individuals. In our assay, purified human NK cells (>95%) from malaria-naïve individuals were co-cultured with 3D7 strain of *P*. *falciparum* infected-RBCs at a starting parasitemia of 0.5%. As shown in Fig 1A, parasitemia reached ~12% by 96 h without NK cells. In the presence of NK cells from one donor, the parasitemia was only ~2%, whereas in the presence of NK cells from a different donor, the parasitemia was ~10%. To compare parasitemia across different experiments with NK cells from different individuals, we calculated percentage of parasitemia reduction as [(parasitemia without NK cells–parasitemia with NK cells) / parasitemia without NK cells] x 100 (see Materials and Methods) and defined individuals whose NK cells reduced parasitemia by more than 50% as responders and those whose NK cells reduced parasitemia by less than 50% as non-responders. On average, responder NK cells (R-NK) reduced parasitemia from 13.5±6.6% to 3.5±1.8% (p<0.0001, n = 19) (Fig 1B), corresponding to a parasitemia reduction of 71±7.4% (n = 19) (Fig 1C), whereas non-responder NK cells (NR-NK) reduced parasitemia from 11.5±5.6% to 9.6±4.9% (p = 0.07, n = 12) (Fig 1B), corresponding to a parasitemia reduction of 25±17% (n = 12, p<0.0001) (Fig 1C). Similar levels of R-NK cells (49.9±3.4%, n = 15) and NR-NK cells (48.4±2.5%, n = 15, p>0.05) were alive after 96 h in the co-cultures (Fig 1D), suggesting that the observed difference in parasitemia is not due to difference in the survival of R-NK and NR-NK cells during the assay.

NK cell activation was assessed by assaying expression of activation markers CD69, CD25, and CD107a. Following co-culture with iRBC for 96 h, NK cells were activated to express CD69 and CD107a (Fig 1E–1G, S1 Table) but the percentages of CD69-positive or CD107a-positive NK cells were significantly higher from responders than non-responders as indicated by log_2_ fold changes (Fig 1I). CD25 was only significantly induced on R-NK cells, but not on NR-NK cells, following co-culture with iRBC (Fig 1E and 1H, S1 Table). No significant changes in expression of natural cytotoxicity receptors NKp30, NKp44, and NKp46 was observed when R-NK and NR-NK cells were co-cultured with iRBC as compared to RBC (S1A–S1C Fig). Of the C-type lectin receptors, both NKG2A and CD94 were significantly upregulated on NK cells following co-culture with iRBC, but there was no difference between responder and non-responder NK cells (S1D and S1E Fig, and S1 Table). No significant difference in expression of NKG2C and NKG2D was observed when R-NK and NR-NK cells were co-cultured with iRBC versus RBC (S1F and S1G Fig). Similarly, no significant difference in expression of the adhesion molecules, such as 2B4, CD36, CD11a, CD18, DNAM-1, and CD2, was observed (S1H–S1M Fig).

NK cell function was examined by assaying for expression of effector molecules granulysin, IFN-γ, and perforin by intracellular staining. R-NK cells significantly upregulated expression of granulysin and IFN-γ, but not perforin, following co-culture with iRBC but not with RBC for 96 h (Fig 1J–1M, S1 Table). No significant increase in expression of granulysin, IFN-γ and perforin were observed when NR-NK cells were co-cultured with iRBC as compared to RBC. Cytokines in culture supernatants at 48 h following iRBC co-culture was measured by multiplex immunoassay. IFN-γ, IL-1β, IL-6, MCP-1, and IL-8 were consistently detected, while IFN-α, TNF-α, IL-10, IL-12, IL-17A, IL-18, IL-23, and IL-33 were below the 2.5 pg/ml detection limit (Fig 1N). Of these cytokines, only IFN-γ levels were significantly higher in R-NK cell cultures (3312±1972pg/ml, n = 19) compared to NR-NK cell cultures (254±177pg/ml, n = 12, p<0.0001).

To verify the observed heterogeneity of NK cell responses, we further tested R-NK and NR-NK cells on three different strains of *P*. *falciparum* parasites–HB2 (isolated in Honduras), T994 (isolated in Thailand) and W2mef (isolated from Indochina). As shown in Fig 1O, regardless of the parasite strain, R-NK cells consistently reduced parasitemia by 50–88%, while NR-NK cells only reduced parasitemia by 6–46% (S2 Table). Collectively, these results show the heterogeneity of NK responses to iRBCs among different individuals in the human population, corroborating with a previous report [20].

### Low NK cell responses are associated with severe malaria

To investigate the clinical relevance of our observation, we purified NK cells from patients with uncomplicated malaria (UM) and severe malaria (SM) and performed *in vitro* iRBC killing assay. NK cells from UM patients reduced parasitemia by 76.0±16% (n = 13), while NK cells from SM patients reduced parasitemia by 60±23% (n = 12) (p = 0.0464) (Fig 1P). The four donors of non-responder NK cells that reduced parasitemia by less than 50% were all from the SM patients. Although the sample size of the current study was small and further studies are required to firmly establish the association between NK cell activity and disease severity, the preliminary results are consistent with an association between low NK cell response and severe malaria [7, 23].

### NK cell responses to iRBCs involve pathogen pattern recognition receptors

To investigate the molecular mechanisms underlying NK cell responses to iRBC between responders and non-responders, we performed genome-wide transcriptional profiling analysis. NK cells from five different responders and five different non-responders were individually co-cultured with either iRBC or RBC for 96 h. NK cells were purified by cell sorting from the four different cultures: R-NK + iRBC (R-iRBC), R-NK + RBC (R-RBC), NR-NK + iRBC (NR-iRBC), and NR-NK + RBC (NR-RBC), for RNA extraction. Gene expression microarray was performed using HumanHT-12 v4 Expression Beadchip (Illumina), which has 47321 probes targeting about 31000 annotated genes.

**Fig 2:**
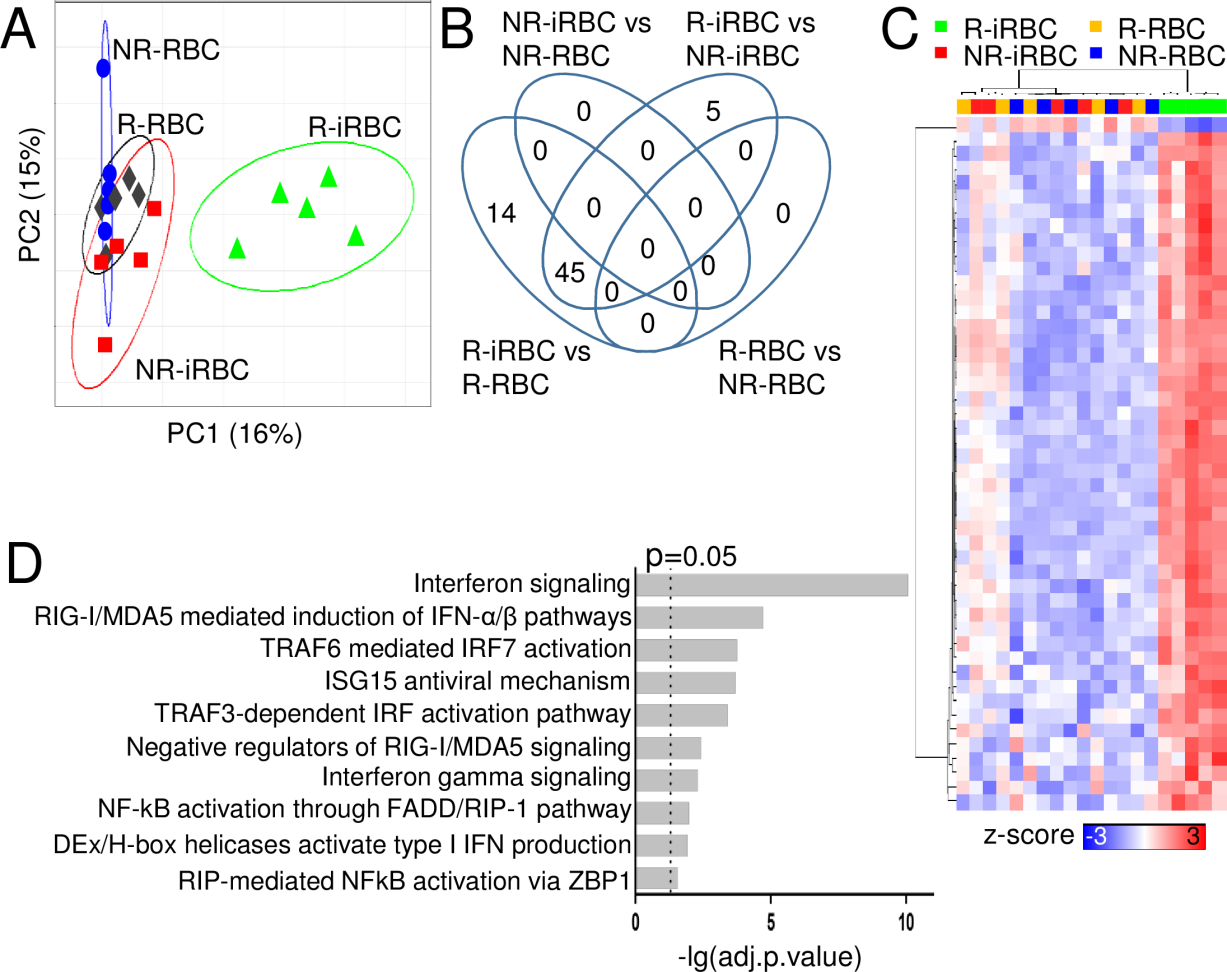
Transcriptional analysis of NK cells in response to RBCs and iRBCs. NK cells from five responders and five non-responders were co-cultured with either iRBCs or RBCs for 96 h. NK cells were purified from the four cultures for RNA isolation, cDNA library construction and microarray analysis. **(A)** sPLS-DA supervised clustering of the indicated conditions. **(B)** Pairwise comparison of all groups. **(C)** Heatmap of expression levels of identified DEGs. Each row represents a gene and each column represents a sample of the indicated group. Hierarchical clustering of columns and rows were performed using Euclidean distance and represented as a dendrogram. The organization and length of the branches in the dendrogram reflect similarities in gene expression profiles. **(D)** Top 10 pathways obtained from functional network-based analysis of the DEGs in R-iRBC samples.

To determine if gross transcriptional differences existed between the groups, we performed sparse partial least square discriminant analysis (sPLS-DA) [24]. Delineation of population relationships with the top two principle components (PC) showed segregation of R-iRBC group from the other three groups (Fig 2A). Next, we performed a comprehensive pairwise comparison of each group to identify differentially expressed genes (DEGs) between groups by linear modeling with a false discovery rate of 5% [25]. A total of 64 DEGs was identified (Fig 2B). 59 DEGs were identified between R-NK cells co-cultured with either iRBC or RBC (R-iRBC vs R-RBC) (S3 Table). 50 DEGS were identified between R-NK and NR-NK cells co-cultured with iRBC (R-iRBC vs NR-iRBC) (S4 Table). Between these two comparisons, 45 DEGs were shared. No DEGs were identified between R-NK and NR-NK cells co-cultured with RBC. Similarly, no DEGs were identified between NR-NK co-cultured with either iRBC or RBC.

To visualize the magnitude of expression of each DEG across samples, z-scores were computed [26]. A heatmap was generated using the derived z-scores with corresponding dendrograms for samples and genes derived via hierarchical clustering using Euclidean distance (Fig 2C). Hierarchical clustering across all samples showed that the samples can be classified into two main groups corresponding to R-iRBC vs the other three groups. Notably, apart from *EIF3L*, whose expression was lower in NK cells from R-iRBC samples, all the other DEGs had higher expression in NK cells from R-iRBC samples.

We performed functional network-based analysis of the DEGs using ConsensusPathDB [27] to identify pathways in which the DEGs could potentially be involved in. The top 10 pathways identified were primarily involved in immune responses and pathogen recognition (Fig 2D). We focused on PRR pathways as they are likely involved in NK cell interactions with iRBCs. The top PRR pathway identified was the RLR signaling pathway. In addition, TRAF3- and TRAF6-dependent pathways were also identified. As these pathways act downstream of Toll-like receptor (TLR) signaling [28], it is possible that TLRs are involved in NK cell responses to iRBCs.

### MDA5 mediates NK cell responses to iRBCs

**Fig 3:**
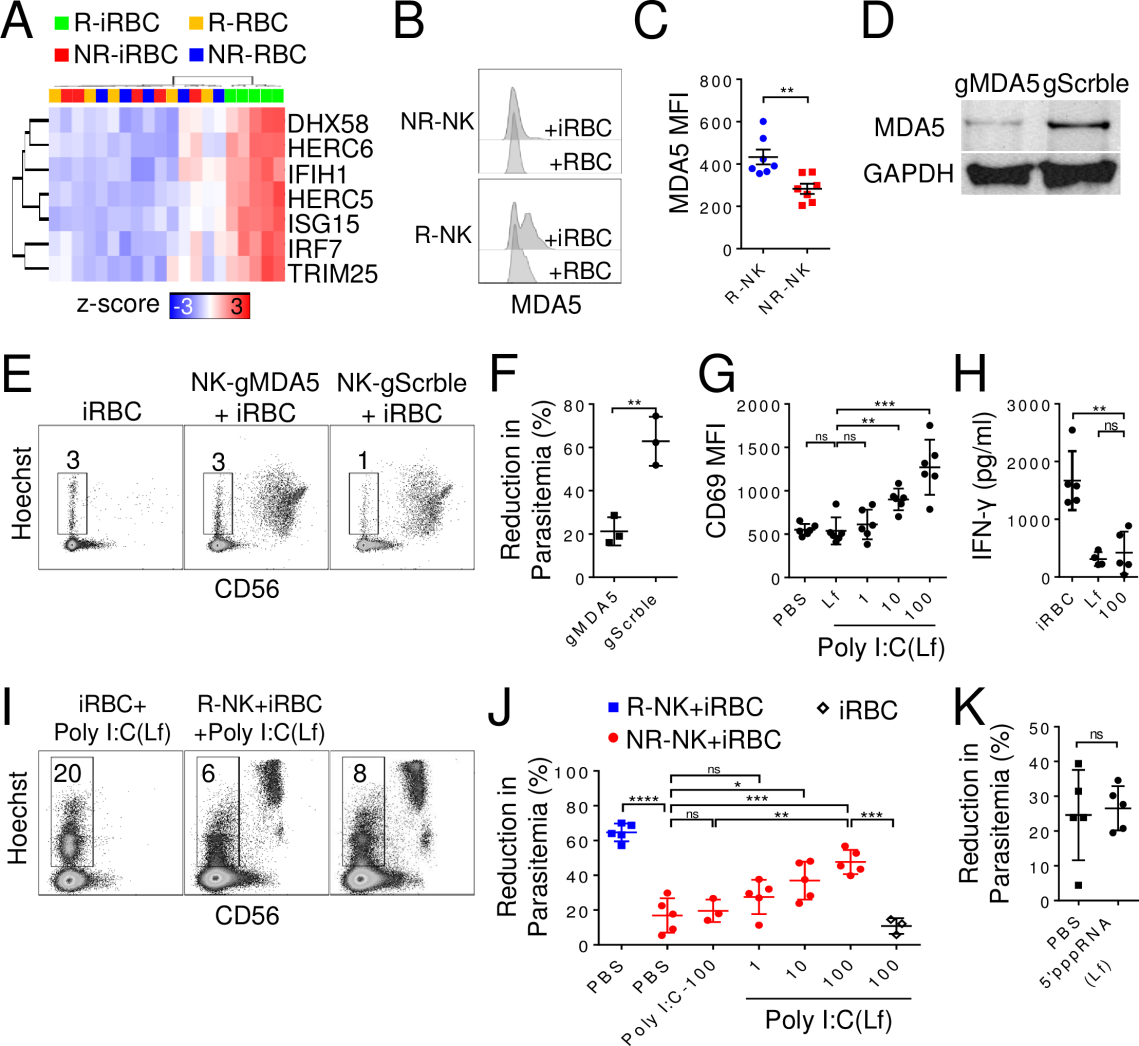
Requirement of MDA5 in NK cell responses to iRBCs. **(A)** Heatmap of DEGs involved in RLR signaling. Each column represents an individual of the indicated group. **(B-C)** NK cells were co-cultured with iRBCs for 96 h, and intracellular staining of MDA5 was analyzed by flow cytometry. Shown are representative histogram **(B)** and MFI **(C)** of MDA5 staining of R-NK and NR-NK cells. **(D)** Western blot of MDA5 and GAPDH levels in R-NK cells transduced with lentivirus expressing CRISPR/Cas9 and a gRNA targeting either MDA5 (gMDA5) or a scramble sequence (gScrble). **(E-F)** Transduced NK cells were co-cultured with iRBCs for 48 h and parasitemia was quantified. Representative Hoechst vs CD56 staining profiles of iRBCs alone, iRBCs co-cultured with R-NK cells with or without MDA5 knockdown **(E)** and comparison of reduction in parasitemia **(F)**. Numbers in E indicate parasitemia. **(G-H)** R-NK cells were co-cultured with either lipofectamine or varying concentrations of lipofectamine-formulated (Lf)-poly I:C for 48 h. Surface expression of CD69 was determined by flow cytometry **(G)**, and IFN-γ secretion was determined by immunoplex assay **(H)**. **(I)** iRBCs were cultured alone or with either R-NK or NR-NK cells in the presence of 1000μg/ml lipofectamine-formulated poly I:C for 96 h. Parasitemia was quantified by flow cytometry. Representative Hoechst vs CD56 staining profiles is shown. **(J)** R-NK and NR-NK cells were co-cultured with iRBCs under the indicated conditions for 96 h, and reduction in parasitemia was quantified by flow cytometry. Poly I:C was used at 100 μg/ml (poly I:C-100). poly I:C (Lf) was used at 100, 10 and 1 μg/ml. **(K)** NR-NK were co-cultured with iRBCs with or without 5’pppRNA (Lf) (10 μg/ml), and reduction in parasitemia was quantified by flow cytometry. Each symbol represents a different individual. Error bars represent mean ± SD. *p<0.05, **p<0.01, ***p<0.001, ****p<0.0001, ns: not significant.

To investigate the role of RLR signaling pathway in NK cell response, we identified the DEGs enriched in the RLR signaling pathway. Expression levels of these enriched DEGs were all considerably higher in the R-iRBC group compared to the other three groups (Fig 3A). Of the three known RLR (RIG-I, MDA5, and LGP2) in cytosolic RNA sensing, only *IFIH1* transcript, which encodes MDA5, was significantly elevated. MDA5 protein level was also significantly higher in R-NK than NR-NK cells following co-culture with iRBCs for 96 h (MFI: 433±93 vs 283±63, p = 0.004, n = 7) (Fig 3B & 3C).

To determine the requirement of MDA5 in NK cell control of parasitemia, we knocked down MDA5 in primary NK cells from responders by lentiviral expression of CRISPR/Cas9 and a guide-RNA targeting either MDA5 (gMDA5) or a scramble sequence (gScrble). After 2 weeks of puromycin selection, MDA5 protein level was reduced by ~70% in NK cells transduced with gMDA5 as compared to NK cells transduced with gScrble by Western blot analysis (Fig 3D). Following co-culture of transduced NK cells with iRBCs for 48 h, MDA5-knocked down NK cells reduced parasitemia by 21±7% (n = 3) whereas gScrble-transduced NK cells reduced parasitemia by 63±11% (n = 3, p<0.01) (Fig 3E & 3F), suggesting a requirement for MDA5 in mediating NK cell responses to iRBCs.

As a complementary approach, we tested the effect of the MDA5 agonist, lipofectamine-packaged poly I:C on NK cells [29]. As shown in Fig 3G, at lipofectamine-poly I:C concentrations of 10 μg/ml (900±125, n = 6, p = 0.0012) and 100 μg/ml (1271±317, n = 6, p = 0.0005) CD69 expression on NK cells was significantly increased compared to lipofectamine treated controls (538±156, n = 6). However, despite the increase in CD69 expression, no significant increase in IFN-γ secretion was detected at 100 μg/ml lipofectamine-poly I:C (421±366, n = 5, p = 0.58) as compared to lipofectamine treated controls (310±120, n = 4) (Fig 3H). We further evaluated whether NR-NK cell responses to iRBCs could be restored by activating MDA5. Indeed, lipofectamine-packaged poly I:C enhanced NR-NK cell responses to iRBCs in a dose-dependent manner, with significant parasitemia reduction observed at 10 μg/ml (37±11%, n = 5, p = 0.02) and 100 μg/ml (48±7%, n = 5, p<0.005) (Fig 3I and 3J), whereas poly I:C without lipofectamine-formulation did not. As a control, lipofectamine-formulated 5’pppRNA, which activates RIG-I, did not induce any significant reduction of parasitemia (Fig 3K).

**Fig 4:**
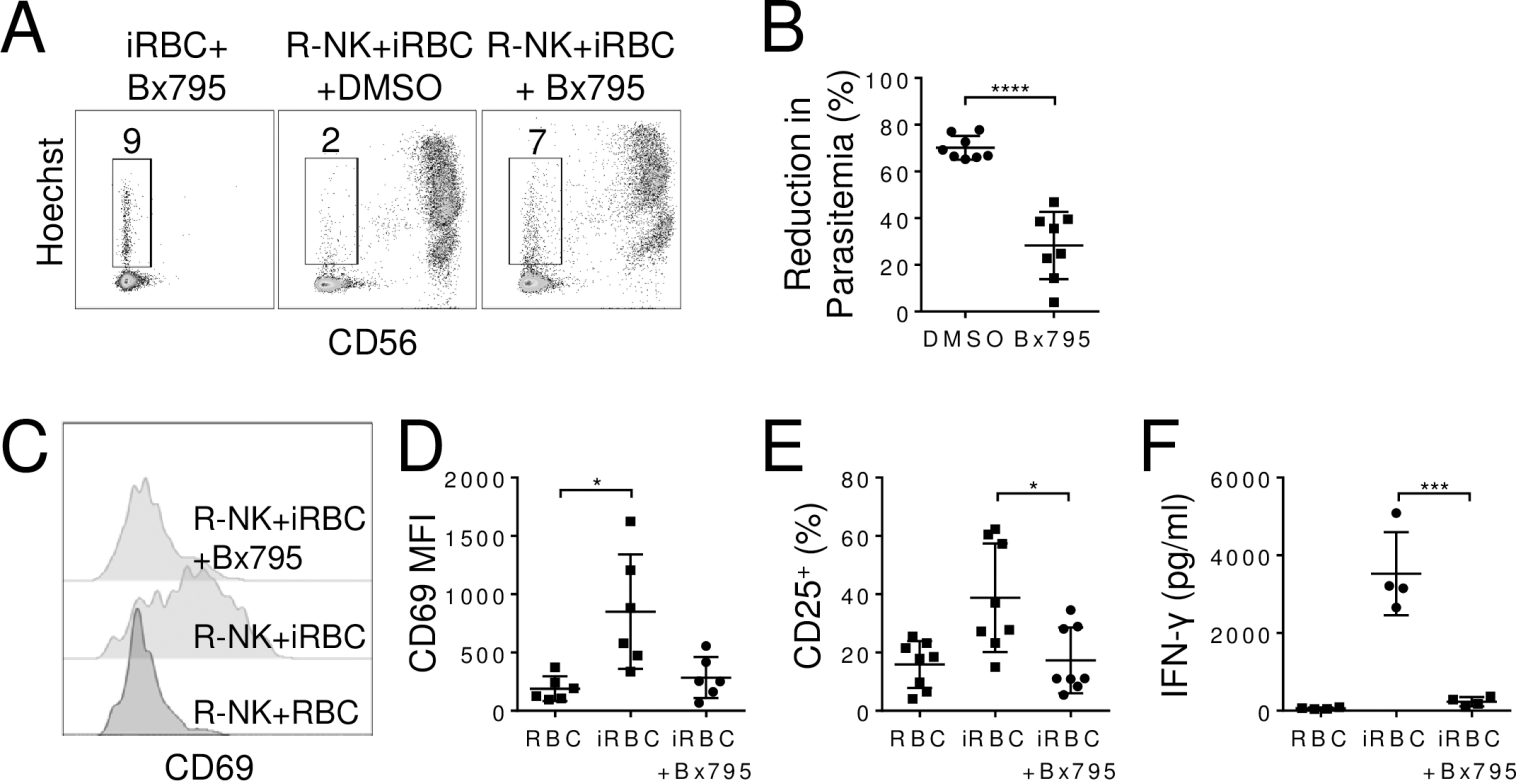
TBK1/IKKε mediates NK cell responses to iRBCs. R-NK cells were co-cultured with iRBCs in the presence or absence of TBK1/IKKε inhibitor Bx795 (10 nM) for 96 h and parasitemia was quantified by flow cytometry. Representative Hoechst vs CD56 staining profiles of indicated cultures. Numbers indicate parasitemia **(A)**. Comparison of reduction in parasitemia **(B)**. Representative histograms of CD69 expression **(C)** and comparison of CD69 MFI **(D)** on NK cells from the indicated co-cultures at 96 h. **(E)** Quantification of the percentage of CD25-positive NK cells after co-culture for 96 h. **(F)** IFN-γ levels in the supernatants of indicated co-cultures after 96 h. Each symbol represents an individual. Error bars represent mean ± SD. *p<0.05, ****p<0.0001.

We also examined the effect of blocking TBK1/IKKε, which is a downstream effector molecule in the MDA5 signaling pathway [30], on NK cell control of parasitemia. R-NK cells were co-cultured with iRBCs in the presence of a small molecule inhibitor of TBK1/IKKε, Bx795 [31], for 96 h. Compared with 70±5.1% (n = 8) reduction in parasitemia in the absence of the inhibitor, the parasitemia reduction was reduced to 28±14% (n = 8, p<0.001) in the presence of Bx795 (Fig 4A & 4B). Bx795 also inhibited NK cell activation by iRBCs as CD69 expression was significantly reduced from a MFI of 850±491 (n = 6) to 285±176 (n = 6, p = 0.02) (Fig 4C & 4D) and the percentages of CD25 positive NK cells fell from 38.2±19.6% to 17.3±11.3% (n = 8, p<0.001) (Fig 4E). IFN-γ secretion by NK cells was similarly reduced (3527±1070 pg/ml vs 232±120 pg/ml, n = 4, p = 0.01) in the presence of Bx795 (Fig 4F). Together, these results show that activation of MDA5 is required for NK cell responses to iRBCs.

### Microvesicles from iRBCs activate NK cells via RLR pathway

**Fig 5:**
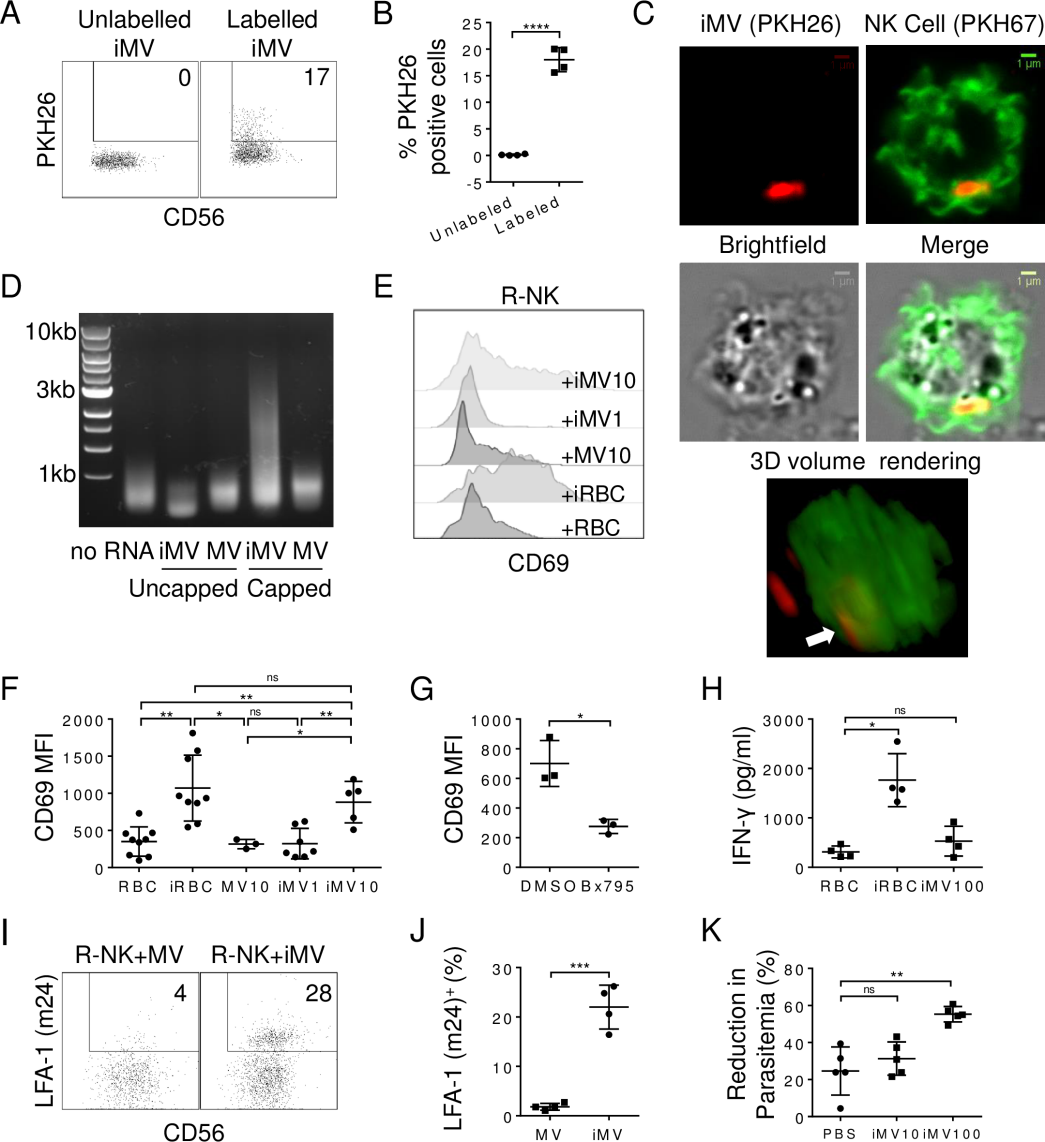
Microvesicles derived from iRBCs prime NK cells. **(A-B)** iMVs (1 μg/ml) were either labeled or not labeled with PKH26 and then cultured with R-NK cells for 24 h. Shown are representative PKH26 vs CD56 staining profiles. Numbers indicate the percentage of positive cells in the gated region **(A)**. Comparison of percentages of PKH26-positive NK cells in cultures with unlabeled or labeled iMV **(B)**. **(C)** Confocal microscopy of PKH67-labelled R-NK cells after 24 h incubation with PKH26-labeled iMVs. Shows are representative images of an NK cell with an internalized iMV. Z-stack of 1 μm slices were captured and 3D volume rendering was performed to demonstrate the location of the internalized iMV (white arrow). **(D)** Gel electrophoresis of cDNA derived from capped and uncapped RNAs of MVs and iMVs. Size markers are labeled on the left. No RNA: no RNA was added during the reverse transcription. **(E-F)** Representative CD69 histograms of R-NK cells cultured with RBC (NK+RBC), iRBCs (NK+iRBCs), MVs at 10 μg/ml (NK+MV10), iMVs at 1 (NK+iMV1) and 10 μg/ml (NK+iMV10) **(E)** and comparison of CD69 MFI on R-NK cells from different donors in the indicated culture conditions **(F)**. **(G)** Comparison of CD69 MFI on R-NK cells cultured with iMVs (at 10 μg/ml) in the presence of DMSO (control) or Bx795 (10 nM). **(H)** IFN-γ levels in the culture supernatants of R-NK cells cultured with RBCs, iRBCs, and iMVs at 100 μg/ml for 48 h. **(I-J)** R-NK cells were cultured in the presence of MVs or iMVs at 10 μg/ml for 96 h, and LFA-1 activation was assessed using m24 antibody. Shown are representative LFA-1 (m24) vs CD56 staining profiles of NK cells **(I)**, and comparison of percentages of LFA-1 (m24)-positive NK cells **(J)** in the indicated conditions. **(K)** Comparison of parasitemia reduction when NR-NK cells were co-cultured with iRBCs without iMVs, with 10 μg/ml iMVs (iMV10) or 100 μg/ml iMVs (iMV100) for 96 h. Each symbol represents a different individual. Error bars represent mean ± SD. *p<0.05, **p<0.01, ***p<0.001, ****p<0.0001, ns: not significant.

MDA5 is a member of cytosolic RLR involved in RNA sensing. To identify the trigger for MDA5 signaling in R-NK cells, we focused on RNA-containing microvesicles [32]. It had been shown that iRBCs actively release microvesicles, especially late during schizont-stages [33]. These microvesicles carry not only parasite-derived proteins [33] but also parasite DNA and RNA [43]. We reasoned that microvesicles could deliver parasite RNAs to the cytosol of NK cells to activate MDA5, similar to lipofectamine-packaged Poly I:C. To test this hypothesis, we purified microvesicles from RBC and iRBC cultures and referred to them as MVs or iMVs, respectively. iMVs were labeled with a lipophilic dye PKH26 and added into R-NK cell culture at 1 μg/ml. After 24 h, 18.0±2.3% of NK cells were positive for PKH26 (p<0.0001, n = 4) (Fig 5A & 5B). To further prove the uptake of iMVs by R-NK cells, we stained R-NK cell membrane with a green lipophilic dye PKH67 and incubated them with PKH26-stained iMVs for 24 h. Confocal microscopy and z-stack 3D volume rendering showed that the PKH26-stained iMV (white arrow) was located within the confines of the PKH67-stained NK cell membrane (Fig 5C), suggesting membrane fusion between iMVs and NK cells.

Studies have shown that MDA5 can be activated by single-strand uncapped RNA [34], double-stranded RNA (dsRNA) of 3kb or larger [35], as well as viral mRNA [36]. To determine the presence of large RNA species in iMVs, total RNA was extracted from both MVs and iMVs. The capped RNA and uncapped 5’pppRNA and 5’ppRNA were separated by labeling uncapped RNAs with desthiobiotin followed by streptavidin column [37]. The capped RNA species and uncapped RNA were both reverse transcribed to cDNA, amplified and visualized on an agarose gel. As shown in Fig 5D, very little if any uncapped 5’pppRNA and 5’ppRNA were detected in either MVs or iMVs. In contrast, capped RNA with size as large as 8 kb was readily detected in iMVs but not MVs. Consistent with recent results showing abundant RNA species within iMVs [38, 39], our results show that iMVs contain large RNA species that could be delivered to MDA5 in NK cells.

To determine if iMVs activate NK cells, R-NK cells were cultured with RBCs, iRBCs, MVs, and iMVs for 48 h and NK cell activation was measured by assaying for CD69 upregulation. MFI of CD69 was increased significantly from 310±184 (n = 8) to 902±487 (n = 8, p<0.01) when R-NK cells were co-cultured with RBCs and iRBCs, respectively (Fig 5E & 5F). No significant change in CD69 MFI was observed when R-NK cells were cultured with 10 μg/ml of MVs (316±63, n = 3) or 1μg/ml of iMVs (323±204, n = 7). However, at 10 μg/ml of iMVs, MFI of CD69 was significantly increased to 651±383 (n = 5). The observed activation of NK cells by iMVs was inhibited by Bx795 as CD69 MFI was reduced from 700±155 in the absence of Bx795 to 275±47 in the presence of Bx795 (n = 3, p = 0.04) (Fig 5G). However, iMVs failed to induce significant production of IFN-γ by NK cells even at concentrations of 100 μg/ml (Fig 5H). These results suggest that iMVs can prime, but not fully activate, NK cells through the MDA5-TBK1/IKKε pathway.

We have shown previously that LFA-1 (CD11a/CD18) is involved in NK cell engagement with iRBCs [10]. We determined if iMVs induce a conformational change in LFA-1 from low affinity to either intermediate or high-affinity conformation. R-NK cells were cultured in the presence of either MVs or iMVs at 10 μg/ml for 48 h and NK cells were stained with m24, an anti-LFA-1 antibody that specifically binds to an epitope on LFA-1 that is only exposed in the intermediate and high-affinity state [40]. Significantly higher percentages of NK cells were positive for m24 in the presence of iMVs (22.0±4.4%, n = 4) than in the presence of MVs (1.8±0.7%, n = 4, p<0.0001) (Fig 5I and 5J). Thus, iMVs also induce a conformational change in LFA-1 from a resting to a primed state on R-NK cells.

We also determined if iMVs can improve NR-NK cells to control parasitemia. As expected, incubation of NR-NK cells with iRBCs led to a reduction of parasitemia by 25±13% (n = 5) (Fig 5K). Although no significant increase in parasitemia reduction was observed when 10 μg/ml iMVs were added into the culture of NR-NK cells and iRBCs, a significant increase (55±4%, n = 5, p = 0.001) was detected when 100 μg/ml iMVs was added, suggesting iMVs can enhance killing of iRBCs by NR-NK cells.

## Discussion

NK cells play a critical role in the immediate responses to malaria parasite infection through rapid activation of cytotoxic activities and secretion of cytokines. Poor NK cell cytotoxicity is often associated with acute malaria [7, 9] and the low NK cell cytotoxicity in pregnant women is associated with higher chance of a positive blood smear for malaria parasites [23]. Interestingly, NK cell responses to malaria infection vary significantly in the human population. NK cells from ~30% of malaria naïve individuals do not become activated nor produce much IFN-γ in response to iRBCs [20]. We also observed similar heterogeneity of NK cell responses to iRBCs among malaria naïve individuals. Besides deficiencies in NK cell activation and IFN-γ production, we further show that NK cells from non-responders are unable to control parasitemia *in vitro*. Analysis of NK cells from patients with uncomplicated or severe malaria also suggests an association of non-responding NK cells with severe malaria.

Our transcriptional analysis of NK cells from responders and non-responders sheds light on the possible molecular basis underlying the variations in NK cell responses to malaria infection. We identified a total of 64 DEGs between R-NK cells co-cultured with iRBCs and the other three groups (R-NK cells co-cultured with RBCs, and NR-NK cells co-cultured with either RBCs or iRBCs). Most of these DEGs are involved in pathways in immune responses and pathogen recognition, especially the RLR signaling pathway. MDA5, one of the three RLRs, has been shown to be required for the control of *P*. *yoelii* infection in mice [41], and for recognition of *Plasmodium* RNA by murine hepatocytes and dendritic cells [16]. Consistently, we show that MDA5 knockdown significantly reduced R-NK cell lysis of iRBCs. Inhibition of TBK1/IKKε, an effector kinase downstream of MDA5, also significantly reduced R-NK cell control of parasitemia. Conversely, NR-NK cells were ‘rescued’ by an MDA5 agonist to control parasitemia *in vitro*. These results show that MDA5 is critical for NK cell responses to iRBCs in human and the difference in their expression following exposure to iRBC may be a significant factor contributing to the variations of NK cell responses to malaria infection in the human population. Because NR-NK cell responses to iRBCs can be restored by an MDA5 agonist, our finding also suggests a possible approach to enhance NK cell activity to malaria infection in non-responders.

MDA5 belongs to a class of intracellular RNA sensors and has typically been associated with recognition of RNA viruses [42]. What are the parasite components that are recognized by MDA5? As parasites reside inside RBCs, how do parasite components gain access and activate MDA5 in the cytoplasm of NK cells? iRBCs are known to shed nucleic acid-containing microvesicles as a form of horizontal cell-cell communication [43]. Recently, Sisquella *et al* reported the presence of parasite mRNA species in iMVs and delivery of nucleic acids to activate the STING pathway by fusion of iMVs with macrophages [44]. We show that NK cells can uptake microvesicles from iRBCs. The fusion of microvesicles with the plasma membrane of NK cells likely releases the nucleic acid-cargo into the cytoplasm of NK cells [45, 46], thereby activating MDA5. Consistently, signaling through the downstream effector molecules, such as TBK1/IKKε, lead to activation of NK cells, including expression of CD69, conformation change of LFA-1 to the intermediate/high-affinity state, secretion of IFN-γ and expression of cytotoxicity. Compared to macrophage activation through STING pathway by iMVs, the RLR/MDA5 pathway is required for NK cell activation by iMVs. Thus, our findings identify the parasite components that are recognized by MDA5 and reveal the mechanism by which parasite components gain access and activate MDA5 in the cytoplasm of NK cells.

It is notable that no DEG was identified between unstimulated R-NK and NR-NK cells. Consistently, R-NK and NR-NK cells are of similar differentiation and maturity status (S2 Fig). These results would suggest that prior to exposure to iRBC, there is no gross difference between R-NK and NR-NK cells, and the difference in their ability to control parasitemia likely arises from their responses to iRBC. It has been reported that resting or naïve NK cells exhibit minimal effector functions, such as cell cytotoxicity and cytokine production following stimulation through activation receptors [47] or PRRs [48]. The transition of naïve NK cells to fully activated effector NK cells involve an intermediate primed state [49]. Unlike naïve NK cells, primed NK cells upregulate activation markers, such as CD69. However, unlike effector NK cells, primed NK cells do not exhibit cytotoxicity or secrete IFN-γ. NK cells can be primed by certain subsets of dendritic cells [50], by cytokines, such as IL-18 [51], and by tumor cell lysates [49]. We found that iMVs activates NK cells to express CD69 but not IFN-γ secretion, suggesting that iRBC-derived microvesicles prime NK cells through MDA5.

We have shown previously that NK cells form stable conjugates with iRBCs in an LFA-1-dependent manner before activation and lysis of iRBCs. Induction of the expression of the intermediate/high-affinity LFA-1 by iMVs would strengthen this interaction. The prolonged interaction between NK cells and iRBCs, in turn, could facilitate the directional release of iMVs to NK cells and likely provides additional signals for full activation of NK cells. In this respect, studies have shown that LFA-1 itself is an important NK cell activation receptor, and the ligation of LFA-1 can lead to the acquisition of cytotoxicity [52]. This notion is consistent with the observations that though IL-12 and IL-18 could activate NK cells, production of IFN-γ still requires direct contact with iRBCs [53]. Thus, it would appear that R-NK and NR-NK cells might have a different threshold in the activation of MDA5, and such small difference in MDA5 responsiveness could amplify the effect of iRBC on NK cells, leading to significant differences in the control of parasitemia. Interestingly, polymorphisms in MDA5 have been reported to affect responses to a variety of viruses, including Coxsackievirus [13], Enterovirus 71 [14], and Hepatitis C virus [15]. As far as we know, no MDA5 polymorphism has been reported to affect the course of malaria infection. Based on our result, it would be of significant interest to investigate this possibility in further studies.

In summary, our study identifies how NK cells respond to parasite-infected RBCs and reveal the molecular basis underlying the variations of NK cell responses to malaria infection in the human population.

## Materials and methods

### Peripheral blood mononuclear cell (PBMC) purification

Whole blood was donated by healthy non-malarial immune adult volunteers at the National University Hospital of Singapore Blood Bank and collected in Citrate-Phosphate-Dextrose-Adenine-1 buffer (CPDA-1, JMS). PBMCs were isolated using Ficoll-Paque PLUS (GE Healthcare) as per manufacturer protocol. Purified PBMCs were resuspended in freezing medium (85% fetal bovine serum [FBS, Gibco], 15% dimethyl sulfoxide [DMSO, Sigma]) and stored in liquid nitrogen. RBCs were washed twice in RPMI 1640 (Sigma-Aldrich), diluted 1:1 with collected plasma, and stored at 4°C for malaria culture.

Buffy coat from malaria patients was performed at the Chittagong Medical College Hospital, Bangladesh. Adult patients (>12 years) were enrolled using the modified World Health Organization criteria for severe falciparum malaria, as defined previously [54]. All patients were enrolled < 24 hours after treatment commencement with either parenteral artemisinin or quinine. Severe malaria was defined as any P. falciparum parasitemia in adults, detected by asexual stages on a peripheral blood slide or a positive rapid diagnostic test in combination with one or more: 1) GCS <11; 2) Hematocrit < 20% with parasite count > 100,000/ mm; 3) Jaundice with bilirubin > 2.5 mg/dl with parasite count > 100,000/ mm; 4) Serum creatinine > 3 mg/dL; 5) Hypoglycemia with venous glucose < 40 mg/dL; 6) Systolic blood pressure < 80mmHg with cool extremities; 7) Peripheral asexual stage parasitemia > 10%; 8) Peripheral venous lactate > 4 mmol/L, 9) Peripheral venous bicarbonate < 15 mmol/L; 10) Respiratory insufficiency. Uncomplicated Malaria required the presence of *P*. *falciparum* asexual stages on a peripheral blood-slide or a positive rapid diagnostic test and the absence of any of the complications of severe malaria.

### *P*. *falciparum* culture and infection

P. falciparum strains 3D7 (MRA-102, contributed by Daniel J. Carucci), HB2 (MRA-767, contributed by ATCC), T994 (MRA-153, contributed by David Walliker), and W2mef (MRA-615, contributed by Alan F. Cowman) were obtained through BEI Resources Repository, NIAID, NIH. Parasites were cultured at 2.5% hematocrit using human RBCs in malaria culture media (MCM) [55]. Cultures were incubated in a Heracell 150 incubator (Thermo Scientific) at 37°C in an atmosphere of 5% CO_2_, 3% O_2_ and 92% N_2_. Schizont-stage iRBCs were harvested by centrifugation on a 60% Percoll gradient [55].

Parasitemia was quantified by flow cytometry and Giemsa-staining [55]. Parasite DNA was stained using Hoechst 33342 (20 μg/ml) (Thermo Scientific). To compare parasitemia from different experiments with NK cells from different individuals, we calculated the reduction in parasitemia as follows:

$$\%Parasitemia\;reduction=\frac{{Parasitemia}_{noNK}-{Parasitemia}_{NK}}{{Parasitemia}_{noNK}}\times 100$$

### *In vitro* NK cell assays

NK cells (CD56^+^CD3^-^) were obtained from PBMC or patient buffy coat by negative selection using EasySep Human NK Cell Enrichment Kit (Stemcell Technologies). The purity of NK cells was consistently between 95–100% (S3 Fig). Synchronized schizonts-stage iRBCs at a parasitemia of 0.5% were incubated with NK cells at a ratio of 1:10. The following antibodies from BioLegend were used to quantify expression of the indicated NK cell markers: CD3 (UCHT1), CD56 (HCD56), CD69 (FN50), CD25 (BC96), CD107a (H4A3), NKp30 (P30-15), NKp44 (P44-8), NKp46 (9E2), NKG2A (16A11), NKG2D (1D11), CD94 (DX22), 2B4 (C1.7), CD36 (5–271), CD11a (HL111), CD18 (1B4/CD18), DNAM1 (11A8), CD2 (RPA-2.10), LFA-1 (m24), Perforin (dG9), Granulysin (DH2), and IFN-γ (4S.B3). Anti-NKG2C (#134522) was obtained from R&D Systems. To control for the inter-individual differences and experimental variations log_2_ fold change of the mean fluorescence intensity (MFI) of the NK cell markers was calculated as follows:

$${log}_{2}fold\;change={log}_{2}\frac{{MFI}_{NK+iRBC}}{{MFI}_{NK+RBC}}$$

Poly I:C was obtained from Invivogen (tlrl-pic) and used at 10 μg/ml. The packaging of poly I:C was performed using lipofectamine 2000 (ThermoFisher) as per manufacturer’s protocol. Bx795 (SML0694, from Sigma-Aldrich) was used to inhibit TBK1/IKKε at a concentration of 10 nM. Quantification of cytokines was performed using LEGENDplex Human Inflammation Panel (13-plex) (BioLegend) at 48 h time point.

To generate MDA5 knockout human NK cells, NK cells were isolated from PMBCs and transduced with lentivirus. Lentivirus was produced in H293FT cells using a 3 plasmid system comprising plentiCRISPRv2 (#52961) [56], pVSVG (#8454) and pCMV-dR8.91. gRNA sequence targeting MDA5 was GCGTTCTCAAACGATGGAGA (http://www.e-crisp.org/E-CRISP/) and scrambled gRNA sequence was GCACTACCAGAGCTAACTCA. Transduced NK cells were cultured in selection medium comprising of RPMI, 10% FBS (Gibco), puromycin (2μg/ml) (Gibco), SCF (20ng/ml), Flt-3L (10ng/ml), IL-15 (10ng/ml), IL-7 (20ng/ml), IL-3 (5ng/ml), β-mercaptoethanol (50μM) (Gibco) for 2 weeks. All cytokines were purchased from Peprotech. NK cells were washed thrice to remove selection medium prior to co-culture with iRBCs. Western blot of MDA5 and GAPDH was performed using anti-MDA5 (#5321, Cell Signaling Technology) and anti-GAPDH (#5174, Cell Signaling Technology), respectively.

### Flow cytometry

Cells were stained with the indicated antibodies for 15 mins at 4°C in flow buffer (0.2% bovine serum albumin [BSA, Sigma], 0.05% sodium azide [Sigma] in PBS). For intracellular staining, brefeldin A (final concentration 5 μg/ml) was added to the culture 6 h before cell harvesting and staining. Cells were fixed in 4% paraformaldehyde in PBS at 4°C for 30 min, followed by permeabilization in perm buffer (0.1% saponin in PBS) at 4°C for 30 min. Antibody staining was then performed in perm buffer at 4°C for 30 min. Stained samples were analyzed on a BD LSR II flow cytometer, and data analyzed by FACS Diva (BD Biosciences) or FlowJo (TreeStar). Isotype-matched control antibodies were used for all fluorochrome-isotype combinations.

### Imaging

Culture chambers were fabricated from Polydimethylsiloxane (PDMS) using Sylgard 184 Silicone elastomer kit (Sigma-Aldrich), cured overnight in a 70°C oven, and cleaned with a plasma cleaner (Harrick Plasma). Culture chambers were then mounted onto Menzel-Gläser 22×60 mm #1 glass coverslips at 70°C. Live-cell imaging was performed on an inverted Olympus IX71 fitted with an Olympus Planapo 60×/1.4 oil lens. Microscope stage was equipped with a Tokai Hit INU Live Cell Microscope Chamber, where the temperature was maintained at 37°C in an atmosphere of 5% CO_2_, 3% O_2_ and 92% N_2_. Images were acquired using a Hamamatsu ORCA-ER (C4742-80-12AG) CCD camera and analyzed using ImageJ.

### Microvesicle isolation

Microvesicles were concentrated using the method described by Mantel *et al* [33]. Briefly, iRBCs or RBC culture supernatants were first centrifuged at 10000 g for 15 mins. Microvesicles were then pelleted from the resultant supernatants at 100000 g for 1 hr using a Ti70 rotor on an Optima L-100K ultracentrifuge (Beckman Coulter). The resultant pellet was resuspended in 2 ml PBS, layered over 10–70% sucrose cushion and centrifuged at 10000 g for 16 h using an SW28Ti rotor. Microvesicles were collected at the interface between 50% and 60% sucrose. Labeling of MV was achieved using either PKH26 or PKH67 (Sigma-Aldrich), as per manufacturer protocol with minor modification. Post-labelling, excess dye was absorbed and washed away with 1% BSA solution 3 times. Microvesicles were then concentrated in a 10 kDa Amicon Ultra-0.5 mL (Merck Millipore) filter. MV concentration was estimated using Nanodrop (Thermo Scientific).

Total RNA from microvesicles was extracted using Trizol (Thermofisher) and capped with desthiobiotin-GTP (NEB) using vaccinia virus mRNA capping enzyme (NEB) as per manufacturer’s protocol. Desthiobiotin-GTP capped RNA was bound to streptavidin column (NEB) and competitively eluted using free biotin (5 mM) (Sigma). Flow-through, containing originally capped RNAs, and eluted RNA species, containing originally uncapped RNAs but *in vitro* capped RNAs, were then reverse transcribed to cDNA with random primers, amplified (Clontech) and visualized on a 1% agarose gel.

### RNA transcriptional analysis

NK cells (1x10^6^) were co-cultured with iRBCs (1x10^5^) or the same number of RBCs as described above. After RBCs were lysed using ACK lysis buffer (Thermo Scientific), live NK cells were purified by cell sorting for RNA extraction by TRIzol (ThermoFisher). Extracted RNA was then processed at DUKE-NUS Genome facility. RNA quality was assessed by electrophoretic assay on the Agilent 2100 Bioanalyzer using a Nano chip (Agilent RNA 6000 Nano chip). 500ng of total RNA with a RIN value of >7.0 was used for biotin-labelling using Illumina TotalPrep RNA Amplification Kit (Ambion Inc.) to generate complementary RNA (cRNA). 750ng of biotin-labeled cRNA was then hybridized on the Illumina HumanHT-12 v4 Beadchip according to Manufacturer’s instruction (Illumina, Inc., San Diego, CA). Beadchips were washed and stained according to manufacturer’s instruction and scanned using Illumina BeadArray Reader (Illumina, Inc., San Diego, CA).

Microarray data were analyzed using R [57]. BeadChip data was converted to an expression set, variance stabilized and quantile-normalized using the lumi package [58]. Differentially expression genes (DEG) were identified via linear modeling using the limma package [25], with a false discovery rate (Bonferroni correction) of 0.05. Pathways were generated using ConsensusPathDB (http://cpdb.molgen.mpg.de) and visualized with PathVisio [59] using WikiPathways [60]. Heatmaps were made with GENE-E (https://software.broadinstitute.org/GENE-E/). Transcriptomic data have been deposited in the ArrayExpress database at EMBL-EBI (https://www.ebi.ac.uk/arrayexpress/experiments/E-MTAB-6574/) under accession number E-MTAB-6574.

### Statistical analysis

Data are presented as the mean and standard deviation (SD). Differences between paired samples were analyzed using a paired t-test, while unpaired samples were analyzed with Student’s t-test. A P value of < 0.05 was considered statistically significant. All calculations were performed using the GraphPad 6.01 software package.

### Ethics statement

Whole blood was donated by healthy non-malarial immune adult volunteers at the National University Hospital of Singapore Blood Bank. Informed consents were obtained from all donors in accordance with a protocol approved by the Institutional Review Board of National University of Singapore (NUS-IRB 10–285). Buffy coat from malaria patients was obtained from adult patients (>12 years) at the Chittagong Medical College Hospital, Bangladesh. The study received ethical clearance by the Oxford Tropical Research Ethics Committee, Oxford, United Kingdom (OxTREC) and the local institutional review board, Chittagong Medical College Ethics Committee, Chittagong, Bangladesh. The study was registered under the trial number NCT02451904. As approved by the institutional review boards, fully informed written consent was obtained, including fully written informed consent from the relative or parent/guardian in cases of reduced consciousness and/or age <16 years.

## Supporting information

S1 Fig Variation of NCRs, C-type lectin receptors and adhesion molecules on NK cell following co-culture with iRBCs. Responder (R-) or non-responder (NR-) NK cells were co-cultured with either RBC or iRBC for 96 hrs. Surface expression of the NCRs–NKp30 **(A)**, NKp44 **(B)**, and NKp46 **(C)**; C-type lectin receptors–NKG2A **(D)**, NKG2C **(E)**, NKG2D **(F)**, and CD94 **(G)**; and adhesion molecules– 2B4 **(H)**, CD36 **(I)**, CD11a **(J),** CD18 **(K)**, DNAM-1 **(L)** and CD2 **(M)** were assessed by flow cytometry. Each dot represents a different individual. Joined lines show experimental pair.
(TIF)

S2 Fig Expression of NK cell maturation markers on responder and non-responder NK cells. (A) Live NK cells from 5 responders (green) and 5 non-responder (yellow) were isolated and processed for microarray transcriptomic analyses. Heatmap of gene expression log2-ratios of selected NK cell differentiation markers is displayed. The relative expression values are color-coded: red–high expression, blue–low. **(B-F)** Surface expression of NKG2A **(B)**, CD94 **(C),** CD36 **(D)**, NKp46 **(E)** and CD57 **(F)** on responder (R-) or non-responder (NR-) NK cells. **(G-H)** Percentage of CD56^+^CD16^+^ **(G)** and CD56^+^CD16^-^ **(H)** cells in responder and non-responders. Each dot represents a different individual. Error bars represent mean ± SD. ns: not significant.
(TIF)

S3 Fig Purity assessment of NK cells after negative selection. NK cells were purified from peripheral blood mononuclear cells by magnetic beads negative selection. Purified NK cells were then stained with DAPI, anti-CD3 (UCHT1) and anti-CD56 (HCD56). Singlets were first gated using FSC-H against FSC-A. NK cell population was then selected on SSC-A against FSC-A. Next, DAPI-negative cells were gated. NK cell purity was then assessed on a CD3 against CD56 plot. Shown were plots from 3 different donors. Number indicates the percentage of the gated population.
(TIF)

S1 Table Changes in activation markers, effector molecules, natural cytotoxicity receptors and adhesion molecules on R-NK and NR-NK cells following iRBC co-culture^a^. (PDF)

S2 Table Control of parasitemia across different *P*. *falciparum* strains by R-NK and NR-NK cells^a^. (PDF)

S3 Table List of differentially expressed genes (DEG) in NK cells of responders upon iRBC exposure compared to RBC. (PDF)

S4 Table List of differentially expressed genes (DEG) in R-NK cells versus NR-NK cells following co-culture with iRBC. (PDF)
