# Supplementary figures and images for: Microvesicles from malaria-infected red blood cells activate natural killer cells via MDA5 pathway
Source: PLoS Pathog. 2018 Oct 4;14(10):e1007298. doi: 10.1371/journal.ppat.1007298 (PMC6171940; doi:10.1371/journal.ppat.1007298)

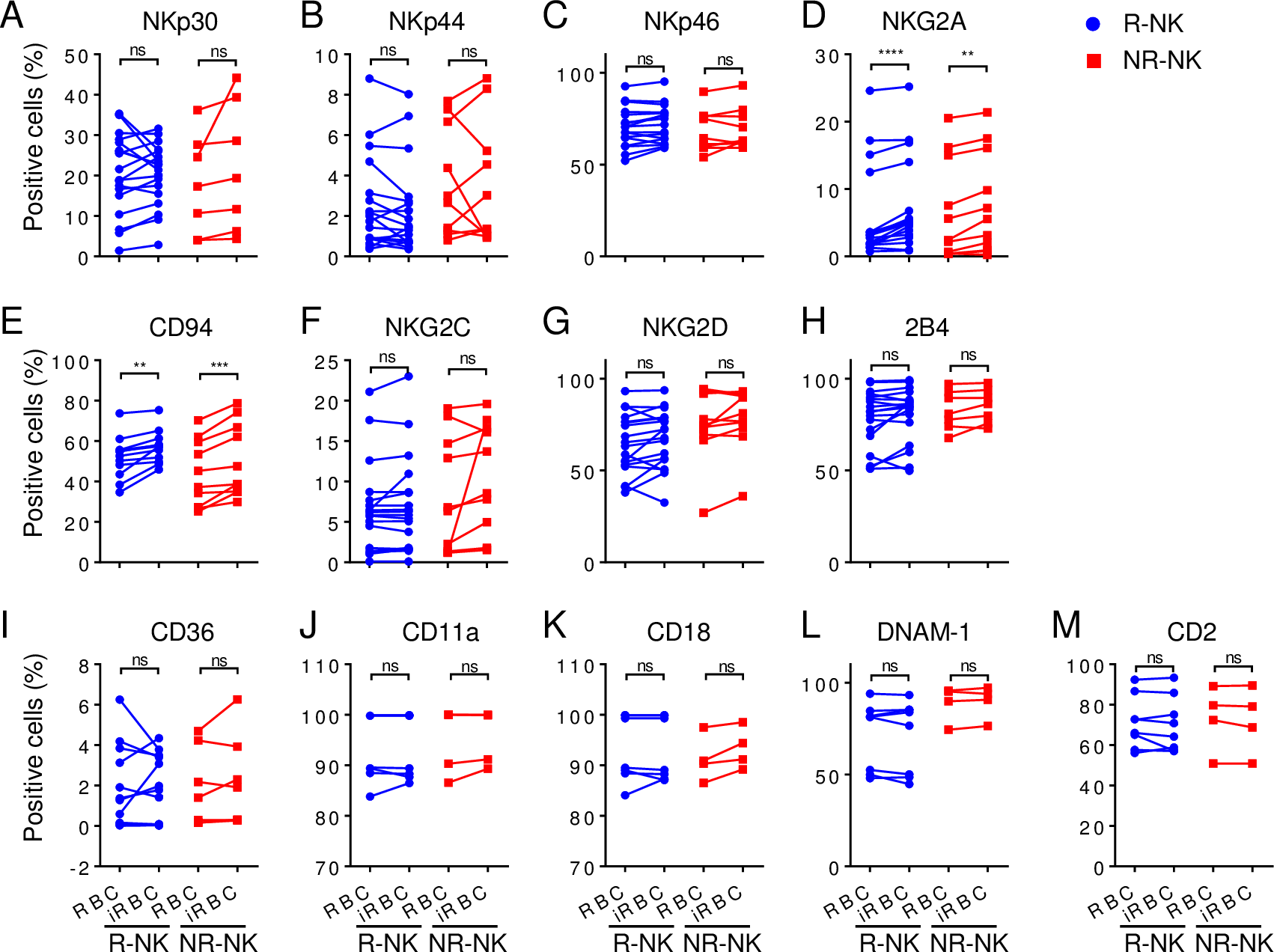

Supplement: S1 Fig — Responder (R-) or non-responder (NR-) NK cells were co-cultured with either RBC or iRBC for 96 hrs. Surface expression of the NCRs–NKp30 (A), NKp44 (B), and NKp46 (C); C-type lectin receptors–NKG2A (D), NKG2C (E), NKG2D (F), and CD94 (G); and adhesion molecules– 2B4 (H), CD36 (I), CD11a (J), CD18 (K), DNAM-1 (L) and CD2 (M) were assessed by flow cytometry. Each dot represents a different individual. Joined lines show experimental pair. (TIF) [file ppat.1007298.s001.tif]

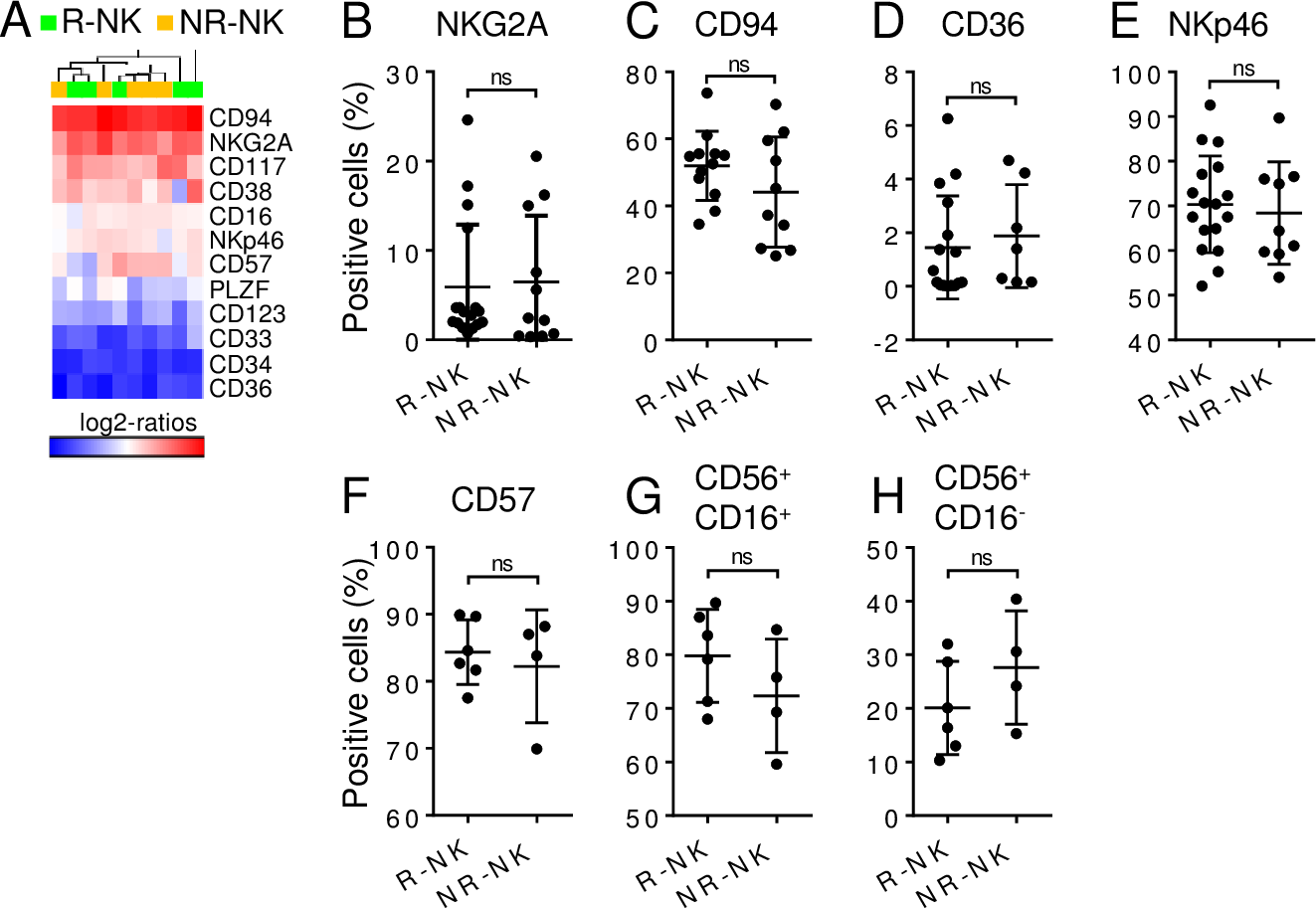

Supplement: S2 Fig — (A) Live NK cells from 5 responders (green) and 5 non-responder (yellow) were isolated and processed for microarray transcriptomic analyses. Heatmap of gene expression log2-ratios of selected NK cell differentiation markers is displayed. The relative expression values are color-coded: red–high expression, blue–low. (B-F) Surface expression of NKG2A (B), CD94 (C), CD36 (D), NKp46 (E) and CD57 (F) on responder (R-) or non-responder (NR-) NK cells. (G-H) Percentage of CD56+CD16+ (G) and CD56+CD16- (H) cells in responder and non-responders. Each dot represents a different individual. Error bars represent mean ± SD. ns: not significant. (TIF) [file ppat.1007298.s002.tif]

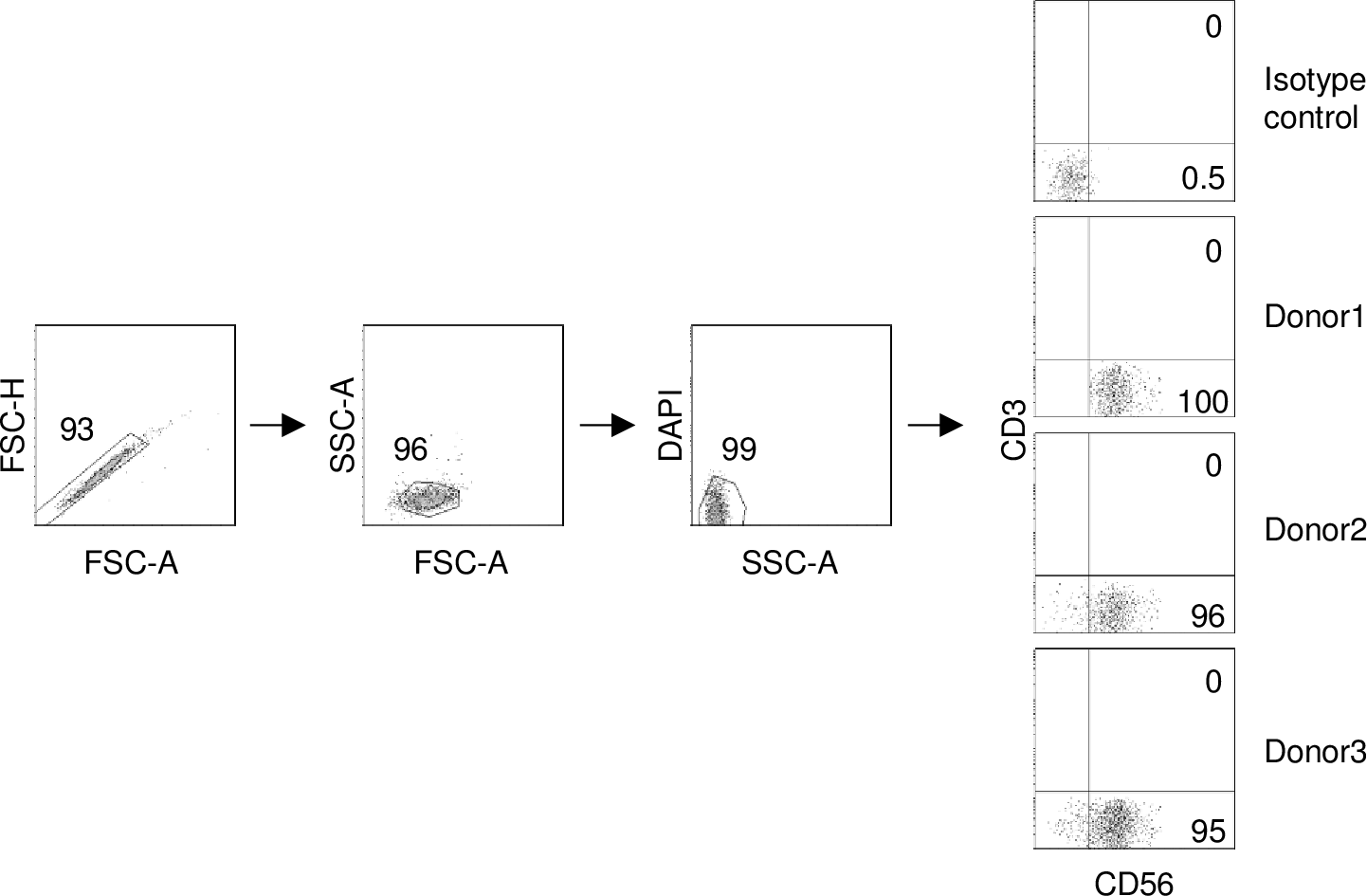

Supplement: S3 Fig — NK cells were purified from peripheral blood mononuclear cells by magnetic beads negative selection. Purified NK cells were then stained with DAPI, anti-CD3 (UCHT1) and anti-CD56 (HCD56). Singlets were first gated using FSC-H against FSC-A. NK cell population was then selected on SSC-A against FSC-A. Next, DAPI-negative cells were gated. NK cell purity was then assessed on a CD3 against CD56 plot. Shown were plots from 3 different donors. Number indicates the percentage of the gated population. (TIF) [file ppat.1007298.s003.tif]
