# Supplementary material for: Microvesicles from malaria-infected red blood cells activate natural killer cells via MDA5 pathway
Source: PLoS Pathog. 2018 Oct 4;14(10):e1007298. doi: 10.1371/journal.ppat.1007298 (PMC6171940; doi:10.1371/journal.ppat.1007298)
Supplement: S1 Table — (PDF) [file ppat.1007298.s004.pdf]

S1 Table. Changes in activation markers, effector molecules, natural cytotoxicity receptors and adhesion molecules on R-NK and NR-NK cells following iRBC co-culture[a].

	R-NK				NR-NK			
**Marker**	**RBC**	**iRBC**	**n**	**p-val[b]**	**RBC**	**iRBC**	**n**	**p-val[b]**
**CD69**	16±11%	44.5±17.4%	18	<0.001	22.7±15.7%	30.7±16.4%	13	0.002
**CD25**	6.4±6.4%	22.4±14.8%	11	0.002	6.8±1.9%	9±4.6%	7	0.185
**CD107**	6.2±3.6%	11.9±5.3%	11	<0.001	7.8±3.5%	9.7±2.7%	7	0.018
**Granulysin**	10.3±6.8%	20.7±14.4%	19	0.001	15.8±11.5%	16.9±11.5%	12	0.359
**IFNg**	1.2±0.8%	5.3±3.9%	19	<0.001	3.7±2.3%	3.9±2.3%	12	0.749
**Perforin**	14.1±13.8%	14±15.8%	17	0.932	11.2±10.5%	15.8±15.5%	7	0.064
**NKp30**	20.5±9.9%	20.3±7.8%	18	0.889	17.8±12.3%	22±15.9%	7	0.158
**NKp44**	2.9±3.4%	2±2%	18	0.054	3.4±2.7%	4.8±4.1%	10	0.167
**NKp46**	63.6±15%	68.4±16%	17	0.034	61.7±21.1%	67.5±15%	9	0.066
**NKG2A**	4.7±7.3%	5.6±8.2%	18	0.032	7.2±9.1%	9.8±10.4%	11	0.019
**NKG2C**	6.7±5.6%	7.1±5.9%	18	0.146	8.4±7.2%	10.8±6.7%	10	0.164
**NKG2D**	64.4±16.3%	67.1±15.8%	18	0.141	74.1±19.5%	77.7±16.9%	10	0.12
**CD94**	52±10.3%	56.3±8%	12	0.011	43.4±19.5%	49.4±18.1%	10	0.04
**2B4**	78.2±15.9%	80.8±14.9%	18	0.092	82.7±10.6%	86.5±7.8%	7	0.033
**CD36**	1.4±1.9%	1.5±1.6%	16	0.893	1.9±1.9%	1.8±2.2%	7	0.84
**CD11a**	93±6.7%	93.1±6.4%	7	0.867	94.2±6.8%	95.1±5.7%	4	0.27
**CD18**	92.9±6.6%	93±6.3%	7	0.857	91.3±4.6%	93.3±4.1%	4	0.051
**DNAM1**	71.7±18.4%	71±19.8%	8	0.528	88.8±10%	89.6±9.2%	4	0.378
**CD2**	71.2±12.9%	70.3±13.7%	8	0.513	73.1±16.3%	72.1±16.5%	4	0.356

[a] Numbers represent the mean±SD of the percentage of NK cells positive for the indicated marker.

[b] p values were calculated using a paired t-test.
