# Supplementary material for: Microvesicles from malaria-infected red blood cells activate natural killer cells via MDA5 pathway
Source: PLoS Pathog. 2018 Oct 4;14(10):e1007298. doi: 10.1371/journal.ppat.1007298 (PMC6171940; doi:10.1371/journal.ppat.1007298)
Supplement: S2 Table — (PDF) [file ppat.1007298.s005.pdf]

S2 Table. Control of parasitemia across different *P. falciparum* strains by R-NK and NR-NK cells[a].

	**R-NK**		**NR-NK**		**t-test**
**Parasite**	**Parasitemia reduction**	**n**	**Parasitemia reduction**	**n**	**p-val[b]**
**3D7**	71±12%	11	25±7.7%	4	<0.0001
**HB3**	63±8.6%	8	27±17%	4	0.0005
**W2mef**	70±10%	8	29±14%	4	0.0001
**T994**	68±12%	6	19±14%	4	0.0003

[a] Numbers represent the mean±SD.

[b] p values were calculated using Student's t-test.
