# Supplementary material for: Microvesicles from malaria-infected red blood cells activate natural killer cells via MDA5 pathway
Source: PLoS Pathog. 2018 Oct 4;14(10):e1007298. doi: 10.1371/journal.ppat.1007298 (PMC6171940; doi:10.1371/journal.ppat.1007298)
Supplement: S3 Table — (PDF) [file ppat.1007298.s006.pdf]

S3 Table. List of differentially expressed genes (DEG) in NK cells of responders upon iRBC exposure compared to RBC.

ACCESSION	SYMBOL	logFC	adj.P.Val#
NM_001548.3	IFIT1	4.426392	6.46E-07
NM_006820.1	IFI44L*	4.401056	0.012936
NM_000619.2	IFNG	4.34097	0.001112
NM_001031683.1	IFIT3	3.462074	0.003645
NM_001549.2	IFIT3	3.308244	4.92E-05
NM_006417.3	IFI44	3.114047	0.007805
NM_022872.2	IFI6	2.981751	0.000146
NM_002462.2	MX1	2.972819	1.41E-05
NM_006187.2	OAS3	2.61062	0.000146
NM_005101.1	ISG15	2.55968	6.46E-07
NM_001032409.1	OAS1	2.463553	1.41E-05
NM_080657.4	RSAD2	2.449901	2.27E-06
BQ437417		2.345501	9.31E-07
NM_022873.2	IFI6	2.344063	0.003147
NM_001032409.1	OAS1	2.325294	4.25E-06
NM_199139.1	XAF1*	2.120073	0.009578
NM_017912.3	HERC6	2.100254	0.00032
NM_001547.4	IFIT2	2.047891	0.007392
NM_199139.1	XAF1*	1.965249	0.035954
NM_016323.2	HERC5	1.894141	9.31E-07
NM_002759.1	EIF2AK2	1.811767	1.41E-05
NM_004029.2	IRF7	1.702383	0.00032
NM_002534.2	OAS1	1.69533	1.41E-05
NM_001031683.1	IFIT3	1.609319	0.00029
NM_004029.2	IRF7	1.599294	0.002649
NM_002346.1	LY6E	1.580198	0.000407

NM_002463.1	MX2	1.530246	0.000208
NM_016817.2	OAS2	1.513264	0.000325
NM_002535.2	OAS2	1.50167	7.06E-05
NM_001100422.1	SPATS2L	1.404516	0.00032
NM_017631.4	DDX60	1.368787	0.003673
NM_033405.2	PRIC285	1.272033	0.003673
NM_001032731.1	OAS2	1.258752	0.011175
NM_024119.2	DHX58	1.246834	0.003307
NM_021105.1	PLSCR1	1.230418	0.000467
NM_207315.2	CMPK2	1.201759	6.46E-07
NM_022168.2	IFIH1	1.188958	0.005372
NM_006074.3	TRIM22*	1.09527	0.046464
NM_012420.1	IFIT5	1.08061	0.003307
NM_030776.1	ZBP1*	1.067086	0.01924
NM_017654.2	SAMD9	1.051433	0.003259
NM_001548.2	IFIT1	1.047809	0.001696
NM_015907.2	LAP3	1.040127	0.003307
NM_016816.2	OAS1	1.00549	0.00032
NM_005082.4	TRIM25	0.991267	0.003801
NM_017554.1	PARP14	0.989533	0.013337
NM_001012967.1	DDX60L*	0.960957	0.019062
NM_016817.2	OAS2*	0.914638	0.002376
NM_004510.2	SP110	0.910701	0.005431
NM_004510.2	SP110*	0.899594	0.046464
NM_015840.2	ADAR*	0.855559	0.01495
NM_001002010.1	NT5C3*	0.787752	0.022796
NM_016410.3	CHMP5	0.780338	0.001092
NM_016410.4	CHMP5*	0.776057	0.008712
NM_017414.3	USP18*	0.775519	0.001092

NM_198576.2	AGRN*	0.724888	0.012562
NM_002468.3	MYD88	0.66141	0.012936
NM_152542.2	PPM1K	0.596809	0.019521
NM_016091.2	EIF3L*	-0.70427	0.009578

# False discovery rate adjusted p-value. Adj. p value < 0.05 is considered as significant.

* Denotes DEGs that are unique to the comparison between R-NK+iRBC and R-NK+RBC.
