# Supplementary material for: Microvesicles from malaria-infected red blood cells activate natural killer cells via MDA5 pathway
Source: PLoS Pathog. 2018 Oct 4;14(10):e1007298. doi: 10.1371/journal.ppat.1007298 (PMC6171940; doi:10.1371/journal.ppat.1007298)
Supplement: S4 Table — (PDF) [file ppat.1007298.s007.pdf]

S4 Table. List of differentially expressed genes (DEG) in R-NK cells versus NR-NK cells following co-culture with iRBC.

ACCESSION	SYMBOL	logFC	adj.P.Va[#]l
NM_001548.3	IFIT1	3.824829	1.51E-05
NM_000619.2	IFNG	3.592687	0.017231
NM_001549.2	IFIT3	2.945553	0.00045
NM_006417.3	IFI44	2.941084	0.021278
NM_001031683.1	IFIT3	2.884268	0.045805
NM_022872.2	IFI6	2.720532	0.000634
NM_002462.2	MX1	2.591678	0.000175
NM_006187.2	OAS3	2.589089	0.000197
NM_080657.4	RSAD2	2.36329	1.30E-05
NM_005101.1	ISG15	2.34414	1.30E-05
NM_001032409.1	OAS1	2.252804	0.000136
NM_022873.2	IFI6	2.180179	0.009531
NM_017912.3	HERC6	2.094705	0.00045
NM_001032409.1	OAS1	2.052004	5.99E-05
BQ437417		1.996662	3.24E-05
NM_001547.4	IFIT2	1.96577	0.017231
NM_002534.2	OAS1	1.810715	1.30E-05
NM_002759.1	EIF2AK2	1.658335	7.58E-05
NM_002463.1	MX2	1.657752	9.01E-05
NM_016323.2	HERC5	1.639694	1.51E-05
NM_004029.2	IRF7	1.471903	0.009483
NM_001031683.1	IFIT3	1.459757	0.001886
NM_016817.2	OAS2	1.455246	0.000755

NM_001100422.1	SPATS2L	1.449311	0.000272
NM_002535.2	OAS2	1.423938	0.000197
NM_033405.2	PRIC285	1.414428	0.001291
NM_017631.4	DDX60	1.402973	0.003902
NM_004029.2	IRF7	1.338296	0.009837
NM_024119.2	DHX58	1.261375	0.003902
NM_021105.1	PLSCR1	1.176727	0.001291
NM_001032731.1	OAS2	1.16718	0.037976
NM_002346.1	LY6E	1.166938	0.024244
NM_001548.2	IFIT1	1.12994	0.000634
NM_022168.2	IFIH1	1.087205	0.021278
NM_207315.2	CMPK2	0.988107	2.89E-05
NM_015907.2	LAP3	0.98403	0.009483
NM_017654.2	SAMD9	0.942738	0.016253
NM_004510.2	SP110	0.94273	0.005263
NM_017554.1	PARP14	0.934579	0.036791
NM_016816.2	OAS1	0.93434	0.00114
NM_144975.3	SLFN5*	0.927353	0.020143
NM_012420.1	IFIT5	0.909867	0.037976
NM_005082.4	TRIM25	0.843358	0.036791
NM_002468.3	MYD88	0.730022	0.006051
NM_016410.3	CHMP5	0.725742	0.003748
XR_016363.2	LOC643384*	0.632803	0.037976
NM_152542.2	PPM1K	0.578964	0.037976
XM_001717833.1	LOC100129334*	0.558653	0.043459
NM_006187.2	OAS3*	0.556268	0.048904

NM_021249.3	SNX6*	0.389579	0.025682

[#] False discovery rate adjusted p-value. Adj. p value < 0.05 is considered as significant.

* Denotes DEGs that are unique to the comparison between R-NK+iRBC and NR-NK+iRBC.
